# Laser speckle contrast imaging with principal component and entropy analysis: a novel approach for depth-independent blood flow assessment

**DOI:** 10.1007/s12200-024-00143-1

**Published:** 2025-01-03

**Authors:** Yu. Surkov, P. Timoshina, I. Serebryakova, D. Stavtcev, I. Kozlov, G. Piavchenko, I. Meglinski, A. Konovalov, D. Telyshev, S. Kuznetcov, E. Genina, V. Tuchin

**Affiliations:** 1 Institution of Physics, Saratov State University, Saratov, 410012 Russia; 2 Scientific Medical Center, Saratov State University, Saratov, 410012 Russia; 3 Laboratory of Laser Molecular Imaging and Machine Learning, Tomsk State University, Tomsk, 634050 Russia; 4 Institute for Bionic Technologies and Engineering, I.M. Sechenov First Moscow State Medical University, Moscow, 119991 Russia; 5 Institute of Biomedical Systems, National Research University of Electronic Technology, Zelenograd, Moscow 124498 Russia; 6 Department of Human Anatomy and Histology, Cytology and Embryology, Institute of Clinical Medicine N.V. Sklifosovsky, I.M. Sechenov First Moscow State Medical University, Moscow, 119991 Russia; 7 Aston Institute of Materials Research, School of Engineering and Applied Science, Aston University, Birmingham, B4 7ET UK; 8 Burdenko Neurosurgery Institute, Moscow, 125047 Russia

**Keywords:** Laser speckle imaging, Speckle contrast, Entropy, Principal component analysis, Blood flow velocity, Vascular imaging

## Abstract

Current study presents an advanced method for improving the visualization of subsurface blood vessels using laser speckle contrast imaging (LSCI), enhanced through principal component analysis (PCA) filtering. By combining LSCI and laser speckle entropy imaging with PCA filtering, the method effectively separates static and dynamic components of the speckle signal, significantly improving the accuracy of blood flow assessments, even in the presence of static scattering layers located above and below the vessel. Experiments conducted on optical phantoms, with the vessel depths ranging from 0.6 to 2 mm, and in vivo studies on a laboratory mouse ear demonstrate substantial improvements in image contrast and resolution. The method’s sensitivity to blood flow velocity within the physiologic range (0.98–19.66 mm/s) is significantly enhanced, while its sensitivity to vessel depth is minimized. These results highlight the method’s ability to assess blood flow velocity independently of vessel depth, overcoming a major limitation of conventional LSCI techniques. The proposed approach holds great potential for non-invasive biomedical imaging, offering improved diagnostic accuracy and contrast in vascular imaging. These findings may be particularly valuable for advancing the use of LSCI in clinical diagnostics and biomedical research, where high precision in blood flow monitoring is essential.

**Graphical Abstract:**

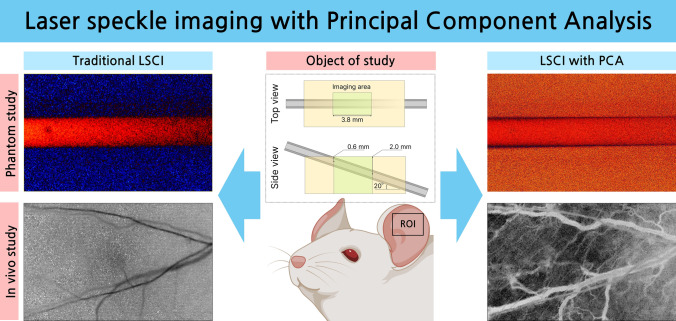

## Introduction

Laser speckle imaging (LSI) is a collection of optical methods based on the analysis of speckle patterns, enabling the visualization of scattering fluid flow in vessels at various depths [1–6], tiny movements [7], and skin hydration detection [8]. LSI setups typically feature a simple design and relatively affordable components, making them suitable for self-assembly or available as ready-to-use commercial devices for laboratory or clinical studies [4, 9, 10]. The ability to perform wide-field (full-field) imaging and the straightforward system setup have led to extensive preclinical and clinical applications of LSI for vascular and tissue perfusion imaging, such as in the skin, retina, brain, and various tumors [1, 2, 11–17]. Among LSI methods, the most common is laser speckle contrast imaging. This method is based on the evaluation of speckle contrast as a quantitative measure of speckle variability, determined by the speed of the observed process [4, 18, 19]. For example, one of the key applications of LSCI in medicine is surgery, where microcirculation imaging plays a crucial role in assessing tissue viability. For example, during organ transplant or tissue graft procedures, real-time blood flow visualization allows surgeons to detect potential perfusion issues in a timely manner and take corrective actions, such as adjusting the flap position or revising the surgical approach [20]. In neurosurgery, LSI is an especially valuable diagnostic tool as it allows the visualization of microcirculation in the brain intraoperatively [1]. Brain perfusion is one of the key parameters determining its functional state, and microcirculatory disorders can lead to irreversible consequences such as stroke or tissue necrosis. Traditional methods for monitoring cerebral blood flow, such as fluorescent angiography, require the introduction of contrast agents and are associated with certain limitations: intolerance to the agent, rapid elimination from the bloodstream, inability to accurately assess blood flow dynamics, and others [17].

LSI is commonly used for mapping and quantitatively assessing relative changes in blood flow in response to interventions and/or external stimuli [15–17]. When biological tissue is illuminated with coherent light, a random speckle-modulated pattern arises due to light scattering. Moving scatterers, such as blood cells, induce phase shifts in the scattered light, while static tissue generates a stable speckle pattern. The statistics of this fluctuating speckle signal provide information about the motion parameters of the scattering particles.

The presence of a static scattering layer above a blood vessel, such as the epidermis or cranial bone, inevitably affects the speckle signal from the vessel. This leads to a reduction in LSI contrast and resolution or may even make blood flow detection difficult [19, 21]. Additionally, changes in the optical properties of the static scattering layer, for instance, due to the application of optical clearing methods to biological tissues, will also reflect in the altered speckle patterns recorded [21, 22]. For accurate assessment of blood flow dynamics, it is necessary to separate changes in speckle patterns caused by static (slowly changing) tissue structures from rapid speckle signal fluctuations resulting from the motion of blood scatterers [23]. For example, when measuring changes in blood flow rate induced by external stimuli, such as the local application of various agents (including optical clearing agents), changes in speckle contrast may be related to both blood flow parameters and the optical properties of the static layer. This complicates the interpretation of results and may lead to incorrect conclusions, where changes in the speckle pattern are perceived as changes in blood flow rate, while in reality, they are caused by alterations in the scattering characteristics of the static layer [6, 13, 21, 22, 24, 25].

Traditionally, methods such as optical clearing and/or surgical interventions, including tissue excision, skull thinning, trepanation, and others, are used to minimize the impact of the static scattering layer above the blood vessels under investigation [19, 21, 22, 26, 27]. Moreover, even with these methods, LSI still provides acceptable resolution and contrast only for superficial vessels, which hardly offers sufficient information for clinical applications.

To overcome these limitations, researchers have proposed several approaches, such as modifying the geometry of the incident and detected laser light, altering LSI optical setups, or developing new algorithms and improving existing ones [19, 23, 28–40]. For instance, studies [19, 29, 30, 39] have shown that altering the direction of the incident light can help extract information from thick tissues. The authors noted that transmissive-detected laser speckle contrast imaging (TrD-LSCI) demonstrates better contrast and resolution than traditional laser speckle contrast imaging with reflective detection (RD-LSCI).

The authors [31] demonstrated the possibility of correcting the influence of the optical properties of surrounding tissues on LSI results. This approach involves the combination of LSI and spatial-frequency imaging for wide-field quantitative hemodynamic visualization, which can correct the effects of changes in optical properties on LSI measurements, including for the quantitative characterization of cerebral blood flow.

The introduction of two polarizers into the optical setup [18, 32] allows for adjusting the sensitivity of LSCI to tissue depth. For example, crossed polarizers placed in front of the laser source and digital camera significantly minimize the influence of the portion of light reflected from the sample surface.

Several approaches have been reported to improve the quality of LSCI by modifying and proposing new algorithms for assessing speckle variability [33–36, 41]. For instance, in [36], the authors proposed using Shannon entropy instead of traditional speckle contrast to assess speckle variability. Miao and co-authors demonstrated that the entropy-based method—laser speckle entropy imaging (LASEI)—exhibits a linear relationship between entropy, fluid flow velocity, and exposure time, and provides higher contrast compared to LSCI.

In studies [37, 38], the authors independently proposed an optical angiography method based on the separation of static and dynamic components of the speckle signal using principal component analysis (PCA) filtering. Using the example of a laboratory mouse’s ear, the superiority of full-scale in vivo optical angiography with PCA filtering over LSCI was demonstrated. Later, Arias-Cruz et al. [23] suggested combining PCA with LSCI to improve the visualization of subsurface blood vessels by separating the static and dynamic components of the speckle signal. The results showed that using PCA in combination with traditional spatial LSCI with time-averaging in the reflective detection mode can enhance the visualization and localization of blood vessels at depths of up to 1 mm. In this study, the authors investigated the effects of the thickness of the static scattering layer above the vessel and the camera exposure time on the quality of LSCI imaging combined with PCA. However, alternative LSCI methods combined with PCA filtering in both reflective and transmissive detection modes were not considered. Additionally, the issue of the proposed method's sensitivity to changes in the scattering fluid's velocity was also left unaddressed.

Given the promising results [37, 38] related to the separation of the speckle signal into contributions from static and dynamic scatterers, as well as the potential of combining PCA filtering with LSI methods [23], this study explores the application of PCA in combination with spatial LSCI with time-averaging (s_avg_-LSCI), temporal LSCI (t-LSCI), spatiotemporal LSCI (st-LSCI), and laser speckle entropy imaging (LASEI) in both reflected-detection (RD) and transmissive-detection (TrD) modes to improve the visualization quality of subsurface vessels by separating static and dynamic components of laser light scattered forward and backward. Additionally, an assessment was conducted of the sensitivity to vessel depth and changes in fluid flow rate for various LSI methods with and without PCA filtering.

## Materials and methods

### Laser speckle contrast imaging (LSCI)

The theory of LSCI is well studied and widely presented in numerous works with practical recommendations and research protocols [6, 18, 19, 35, 42–44]. In short, if the scatterers are mobile, as is the case with erythrocytes moving within blood vessels, the time-integrated speckle pattern, recorded over a finite camera exposure time, becomes blurred. The extent of blurring is quantitatively represented by the parameter termed speckle contrast, and it is calculated as follows:

1 $$\begin{array}{c}LSC\left(x,y\right)={\sigma }_{p}/{\langle I\rangle }_{p},\end{array}$$

where *LSC* is the speckle contrast, and *σ*_*p*_​ and ​⟨*I*⟩_*p*_ are respectively the standard deviation and mean intensity of pixels within a predefined neighborhood *p* around the pixel with coordinates *x, y*. For spatial LSC estimation, the neighborhood *p* represents a square of pixels in the spatial domain, and for spatiotemporal LSC estimation, *p* is a cuboid of pixels in the spatiotemporal domain. The calculation of *LSC*(*x*,*y*) is performed across the entire image using a sliding window.

According to [31], for example, the speckle flow index (*SFI*) is proportional to the linear velocity of fluid flow. *SFI* is calculated using the following simplified equation:

2 $$\begin{array}{c}SFI\left(v\right)\sim \frac{1}{2\cdot T\cdot {LSC\left(v\right)}^{2}}, \end{array}$$

where *T* is the camera exposure time.

### Laser speckle entropy imaging (LASEI)

In LASEI, entropy analysis is used as a local numerical characteristic of speckle variability. During entropy analysis, consecutive intensity values of the same pixel in the camera matrix are used [36], making this method similar to t-LSCI. Entropy is a characteristic measure that describes the degree of image blurring. The entropy of a single pixel is determined by the following formula [45]:

3.1 $$\begin{array}{c}LASE=\sum_{i=0}^{{2}^{b}-1}[{p}_{i}\text{log}{p}_{i} ] ,\end{array}$$

where *LASE* is the entropy value, *b* is the number of bits encoding the pixel intensity values, determined by the camera parameters, and *p*_*i*_​ is the probability of the occurrence of the *i*-th gray level in the selected sequence of frames for a single pixel in the matrix. Where *p*_*i*_ ​ is computed as the ratio of the number of pixels with intensity *i*, denoted as *n*_*i*_, to the total size of the data set *N*.

For a more accurate assessment of Shannon entropy using relatively small data sets, a “balanced estimator” was proposed [36, 44, 45] which is characterized by smaller statistical errors:

3.2 $$\begin{array}{c}LASE\approx \frac{1}{N+2}\sum_{i=0}^{{2}^{b}-1}\left(\left({n}_{i}+1\right)\sum_{j={n}_{i}+2}^{N+2}\frac{1}{j}\right).\end{array}$$

When applying entropy analysis to laser speckle imaging, a limited set of speckle images can lead to significant estimation errors and statistical inaccuracies. Therefore, at least 80 consecutive RSI (raw speckle images) are required for a reliable entropy assessment [36].

According to [35], the entropy parameter has a linear relationship with fluid speed:

4 $$\begin{array}{c}LASE\left(v\right)=-b\cdot v+c ,\end{array}$$

where *b* and *c* are normalization parameters determined empirically. Thus, the dimensionless *SFI* analog for LASEI can be defined as:

5 $$\begin{array}{c}SFI\left(v\right)\sim \frac{1}{LASE\left(v\right)}.\end{array}$$

### Principal component analysis (PCA)

PCA is widely used across various fields of science. It is applied to reduce data dimensionality and extract the most significant components and structures. The application of PCA filtering in the context of LSI is detailed in [23, 37, 38, 46]. In short, in PCA filtering, it is assumed that the camera matrix records a time series of *N* frames—RSI. Each frame consists of a pixel array of size *P* × *Q*. When viewed along the time axis, the recorded sequential speckle signal consists of a low spatial frequency signal from static tissue, a highly fluctuating signal from moving scatterers, and random white noise. Thus, the speckle signal can be expressed in the following matrix form, which contains *N* vectors:

6 $$\begin{array}{c}X={\left[{x}_{1},{x}_{2},\dots ,{x}_{N}\right]}^{\text{T}}={X}_{s}+{X}_{d}+{X}_{w} ,\end{array}$$

where the superscript T denotes the matrix transpose, and *x*_*i*_ is a one-dimensional vector of length *M* = (*P* × *Q*) – a one-dimensional representation of the frame, obtained by filling one row after another, representing the *i*-th frame. Thus, all time points for a single pixel in a 2D speckle image are arranged in one row along *X*. The matrices *Xs*, *X*_*d*_, and *X*_*w*_ of size *M* × *N* represent speckle signals from static scatterers, fluctuating signals from moving scatterers, and white noise, respectively. From a vector space perspective, filtering can be viewed as applying a linear matrix operator to the original speckle signal vector *X*.

Since the blood volume constitutes only about 7% of the total tissue volume in healthy mammals [47, 48], the object of study is globally static over time, and therefore, the RSI exhibit correlations with each other. This correlation is analyzed and extracted using PCA. The calculation of uncorrelated principal component (*PC*) estimates is performed using the following formula:

7 $$\begin{array}{c}PC={\Lambda }^{\text{T}}\cdot \phi ,\end{array}$$

where Λ is the *M* × *M* matrix of orthonormal eigenvectors, and* ϕ* is the matrix obtained after centering the one-dimensional vectors *x*_*i*_ ​ from matrix *X*: ${\phi }_{i}={x}_{i}-\mu \left({x}_{i}\right)$, where *μ*(*x*_*i*_) is the mean of *x*_*i*_. The matrix Λ can be obtained from the following equation:

8 $$\begin{array}{c}C={\Lambda \lambda\Lambda }^{\text{T}} ,\end{array}$$

where *C* is the covariance matrix:

9 $$\begin{array}{c}C={\phi \cdot \phi }^{\text{T}},\end{array}$$

*ϕ* and *ϕ*^T^ represent the centered matrix and its transpose, respectively.

The principal components can be interpreted as speckle pseudo-images that show no correlation with each other. The original data can be fully restored only if the entire set of eigenvectors is used in the inverse transformation:

10 $$\begin{array}{c}X=\left(\Lambda \cdot PC\right)+\mu \left(x\right). \end{array}$$

On the other hand, if only part of the eigenvectors is used in the inverse transformation, it is still possible to construct a new data set (an approximation), in which certain undesirable characteristics can be isolated or removed from the original data.

In studies [37, 38], it is suggested to use only the first principal component to estimate *X*_*s*_​. However, the selection of principal components describing the static part of the signal can be optimized, for example, using the Guttman − Kaiser criterion [23]. The dynamic part of the signal can be defined as:

11 $$\begin{array}{c}{X}_{d}+{X}_{w}=X-{X}_{s}. \end{array}$$

### Optical phantom

Epoxy resin was used as the base for the optical phantom, containing titanium dioxide (TiO_2_) microparticles as scatterers with an average diameter of 100 − 120 nm at a concentration of 0.75 mg/mL. To simulate a vessel, a glass capillary with an inner and outer diameter of 600 and 1000 µm, respectively, was embedded at a 20° angle to the surface of the phantom base. By positioning the glass tube at an angle, we effectively modeled various vessel depths with a gradually changing thickness of the static base of the optical phantom above and below the vessel. A mold was designed and 3D-printed with a height of 4 mm, a width and length of 10 mm, and special slots for placing the glass capillary. The designed mold with the glass capillary was placed on a coverslip to prevent epoxy resin leakage. A pre-prepared epoxy resin solution with a hardener and uniformly dispersed titanium dioxide powder was poured into the prepared mold. After the epoxy resin had fully cured, the optical phantoms were checked for the absence of bubbles, uneven scatterer distribution, and other defects. If visible defects were found, the phantom preparation procedure was repeated. After preparing the optical phantom, the coverslip at the bottom of the phantom was carefully removed.

To model the scattering properties of blood, a 3% aqueous solution of intralipid was used [23], infused at a rate of 1 to 20 mL/h in 1 mL/h increments using a syringe pump SN-50F6 (KRANZ, China). As the fluid flow rate increased, a 90-s pause was introduced between measurements to minimize turbulent effects in the fluid flow due to the rapid increase in infusion speed. Poiseuille’s law was used to convert the volumetric flow rate into linear rate.

### LSI optical system

A coherent light source was used for speckle imaging: a single-mode He–Ne laser HNL210L (Thorlabs, USA) with a wavelength of 632.8 nm and 20 mW power. A laser beam expander was used to uniformly illuminate an area with a diameter of approximately 1.5 cm. The RSI of the surface of the analyzed area were recorded using a monochrome CMOS camera (Thorlabs CS235MU Kiralux series, USA, with a pixel matrix of 1920 × 1200 pixels (2.3 MP), pixel size 5.86 µm × 5.86 µm; 8-bit/pixel) equipped with a 3.7 × magnification LOMO micro-objective with a numerical aperture of 0.11. The speckle size in our LSI system was approximately 12.9 μm. Thus, the ratio of the speckle size to the pixel size of the camera sensor was approximately 2.2. According to [18], this ratio is sufficient for accurate estimation of speckle contrast values. The camera exposure time was set to 1 ms, and the frame rate was 39 frames per second.

**Fig. 1:**
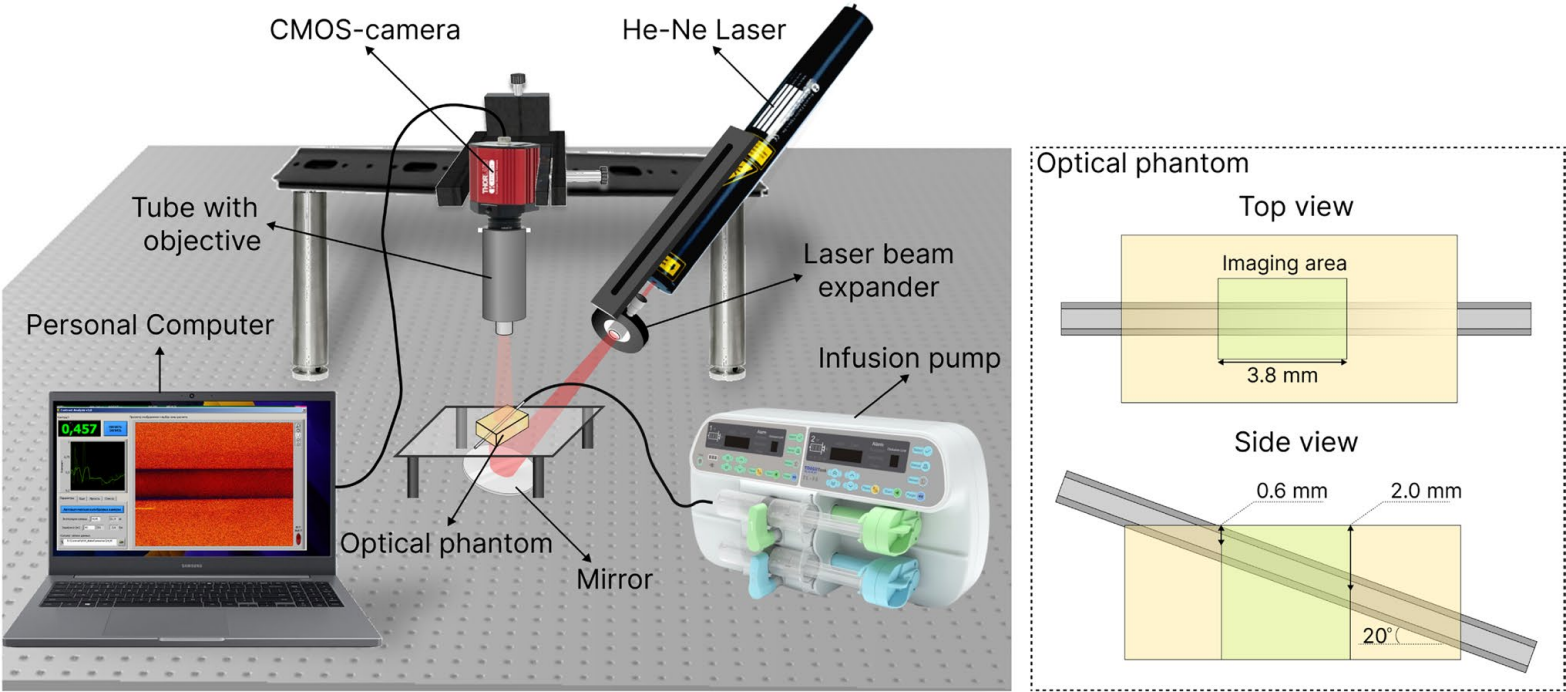
Typical transmissive-detected LSI setup—on the left and the optical phantom schematics—on the right

For the RD mode of recording RSI, the laser with a beam expander was positioned to illuminate the surface of the optical phantom. In the TrD mode, the laser beam was directed through an optical mirror onto the bottom of the optical phantom. A schematic drawing of the setup in the TrD configuration and the optical phantom with the glass capillary is presented in Fig. 1. Measurements in the TrD and RD modes were performed sequentially. First, TrD measurements were taken for the entire range of investigated speeds, followed by RD measurements.

### PCA based filtering

Following the procedure described in Sect. 2.3 and the recommendations in [23, 37, 38], each RSI was transformed into a column vector. The set of consecutive RSI formed a matrix of size *M* × *N*, where *M* = 2,304,000 is the total number of pixels in the camera matrix (1200 × 1920), and *N* = 100 is the number of consecutive RSI. After extracting the principal components, they were divided into two groups (*Static component* and *Dynamic component*) according to the Guttman − Kaiser criterion [23, 49]. The *Static component* group included all *PC*s whose eigenvalues satisfied the condition ${\lambda }_{i}\ge \overline{\lambda }$, where $\overline{\lambda }$ is the mean of all eigenvalues.

After the *Static component* group was formed, the corresponding speckle pseudo-images were reconstructed using Eq. (10). Additionally, the *Original signal* group was created, representing the set of RSI.

### Parameters for calculating LSCI and LASEI

The temporal speckle contrast and entropy were calculated using a time window of 100 frames, while the spatial speckle contrast was calculated using a sliding spatial window of 7 × 7 pixels. The resulting s_avg_-LSC images were averaged over 100 frames, and the spatiotemporal speckle contrast was calculated using a 7 × 7 pixel spatial window and a time window of 100 frames.

**Fig. 2:**
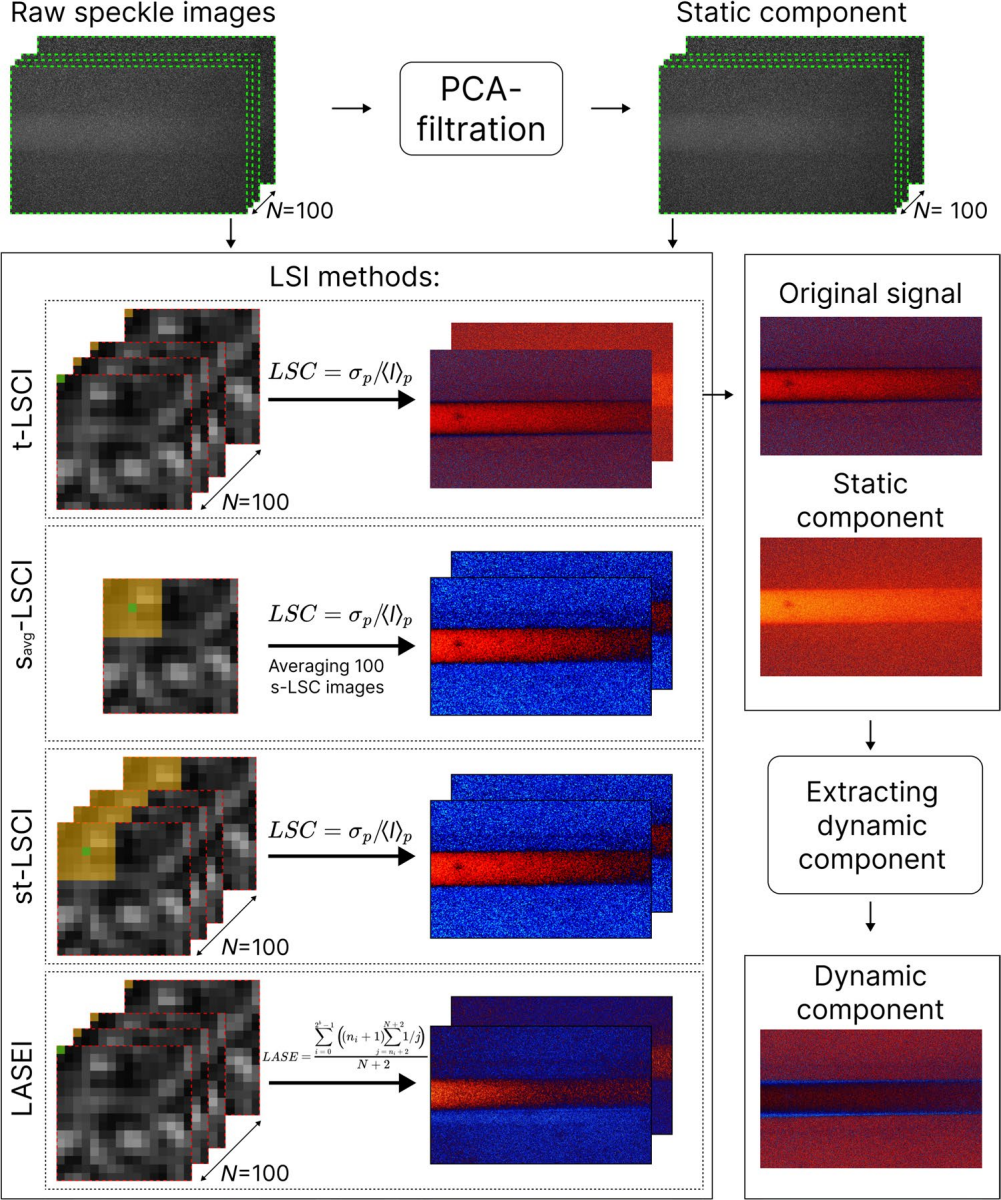
LSI processing pipeline from raw speckle images to 3 groups of LSI-images: *Original signal*, *Static component* and *Dynamic component*. In the LSI methods block on a fragment of raw speckle image orange pixels are used to calculate speckle contrast or entropy for a green pixel

Speckle contrast and speckle entropy images were calculated for the entire range of speeds and for each group of images: *Original signal* and *Static component* obtained for both TrD and RD modes. A new group of images—*Dynamic component*—was formed by subtracting the *Static component* images from the *Original signal* images. Figure 2 shows the processing pipeline from RSI to the three groups of LSI images.

To analyze the sensitivity of LSI methods with and without PCA filtering to vessel depth, the mean *SFI* value over a 100 µm × 100 µm region was calculated for the *Original signal* and *Dynamic component* groups for five equidistant vessel depths using the entire set of images. The resulting set of *SFI*(*v*) for five depths and each LSI method was approximated with a linear function. Then, the deviation of the slope of *SFI*(*v*) for each depth from the average slope (averaged across all depths) was calculated as a percentage. The resulting values were presented in matrix form to visually assess the sensitivity of LSI to vessel depth. The rows of this matrix corresponded to LSI methods, and the columns to the depths of the glass capillary.

To analyze the sensitivity of LSI methods to changes in blood flow rate, the Pearson linear correlation coefficient between *SFI*(*v*) and the linear blood flow speed (*v*) was calculated. The resulting values were also presented in matrix form for clarity.

A custom software package was developed for RSI processing and analysis, written in Python.

### In vivo study

In this study, a single ear of a BALB/c mouse weighing 25 g was used for in vivo demonstration to assess the effectiveness of the LSCI and LASEI methods with PCA for blood flow visualization. The choice of a single sample was due to the aim of the study, which focused on demonstrating the reproducibility and reliability of the proposed method rather than biological variability. Measurements were repeated multiple times (*n* = 20) on the same sample to ensure stability and repeatability of the results. This approach is based on previous studies where similar techniques were applied for blood flow assessment and vascular visualization [50–52]. In this study, animal experiments were conducted in accordance with ethical standards. The experiments involving the mouse ear were approved by the Ethics Committee of Saratov State Medical University (Protocol No. 11, dated August 7, 2022).

## Results

### Comparison of LSI methods with and without PCA filtering

**Fig. 3:**
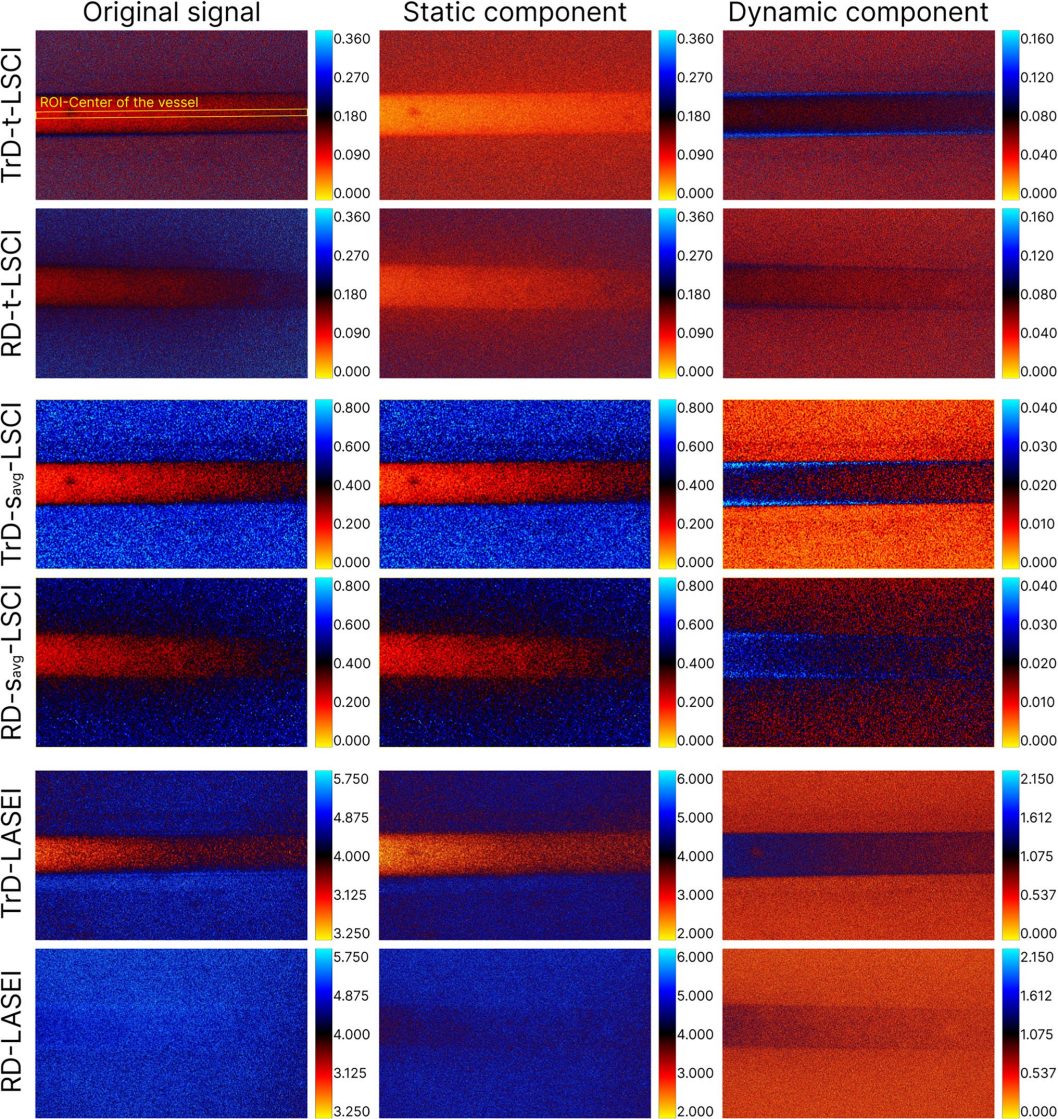
Color maps of speckle contrast and entropy values obtained using various laser speckle imaging (LSI) methods in transmissive (TrD) and reflective (RD) laser detection modes at a linear scattering fluid velocity of 15.72 mm/s. The first column (*Original signal*) represents the results of RSI processing, containing both static and dynamic components of the speckle signal. The second column (*Static component*) corresponds to the results of processing the *Static component* of the speckle signal. The third column (*Dynamic component*) reflects the *Dynamic component* associated with the movement of scattering particles. The color palette was chosen for optimal representation of the dynamic range for each LSI method in TrD and RD modes. The region of interest, marked with a yellow rectangle, has a width of 100 µm and a length of 4000 µm, corresponding to the central part of the glass capillary

Figure 3 illustrates the color maps obtained from processing the speckle image set using various LSI methods in TrD and RD detection modes at a linear scattering fluid speed of 15.72 mm/s, corresponding to flow in arterioles and venules [23, 53, 54]. The first column (*Original signal*) presents the results of RSI processing, where both static and dynamic signal components are present, corresponding to traditional LSI methods. The second column (*Static component*) illustrates the results after extracting the *Static component* of the speckle signal and applying LSI methods. Here, fluctuations caused by moving particles have been removed through PCA filtering. The third column (*Dynamic component*) represents the difference between the first and second columns and corresponds to the Dynamic *component* reflecting changes caused by moving scatterers. The color palette was chosen to effectively capture the dynamic range for each LSI method in TrD and RD modes. In the images for the *Original signal* and *Static component* groups, it is easy to observe that as the thickness of the scattering layer increases (from 0.6 to 2 mm), the speckle contrast and entropy values in the region of the glass capillary also increase. Comparing the recording methods in TrD and RD modes, it is noticeable that the temporal and spatial speckle contrast values in the glass capillary region decrease faster with increasing vessel depth in RD mode. At depths of around 1.8–2 mm, LSCI images of the vessel become blurred and blend into the background. However, in transmissive detection, LSCI images of the vessel remain distinguishable throughout the entire imaging area. This effect may be due to the fact that TrD-LSI is primarily formed by forward-scattered light, while traditional RD-LSCI relies on backscattered light [19]. Thus, it can be concluded that TrD-LSCI methods are more effective for deep visualization compared to RD-LSCI, although this may only hold true for certain thicknesses.

Notably, the speckle contrast values for the *Dynamic component* in the glass capillary region are higher than the background. This is atypical for LSI, as static scattering backgrounds usually exhibit higher speckle contrast or entropy values. This effect is caused by the fact that in the *Dynamic component* group, the signal from the static scattering base surrounding the vessel is either absent or minimized. On one hand, this leads to lower contrast in the vessel image at high flow rates, but on the other hand, it minimizes the sensitivity of LSI to vessel depth and allows for a more confident determination of scattering fluid velocity. It may seem that with the complete removal of the speckle signal from the *Static component* of the optical phantom, the background values for the *Dynamic component* group should approach zero. However, during laser light scattering by both dynamic and static scatterers, there is a fraction of multiply scattered light that, due to consecutive scattering events, also reaches the camera matrix and carries information about the scatterers (including their movement velocity).

**Fig. 4:**
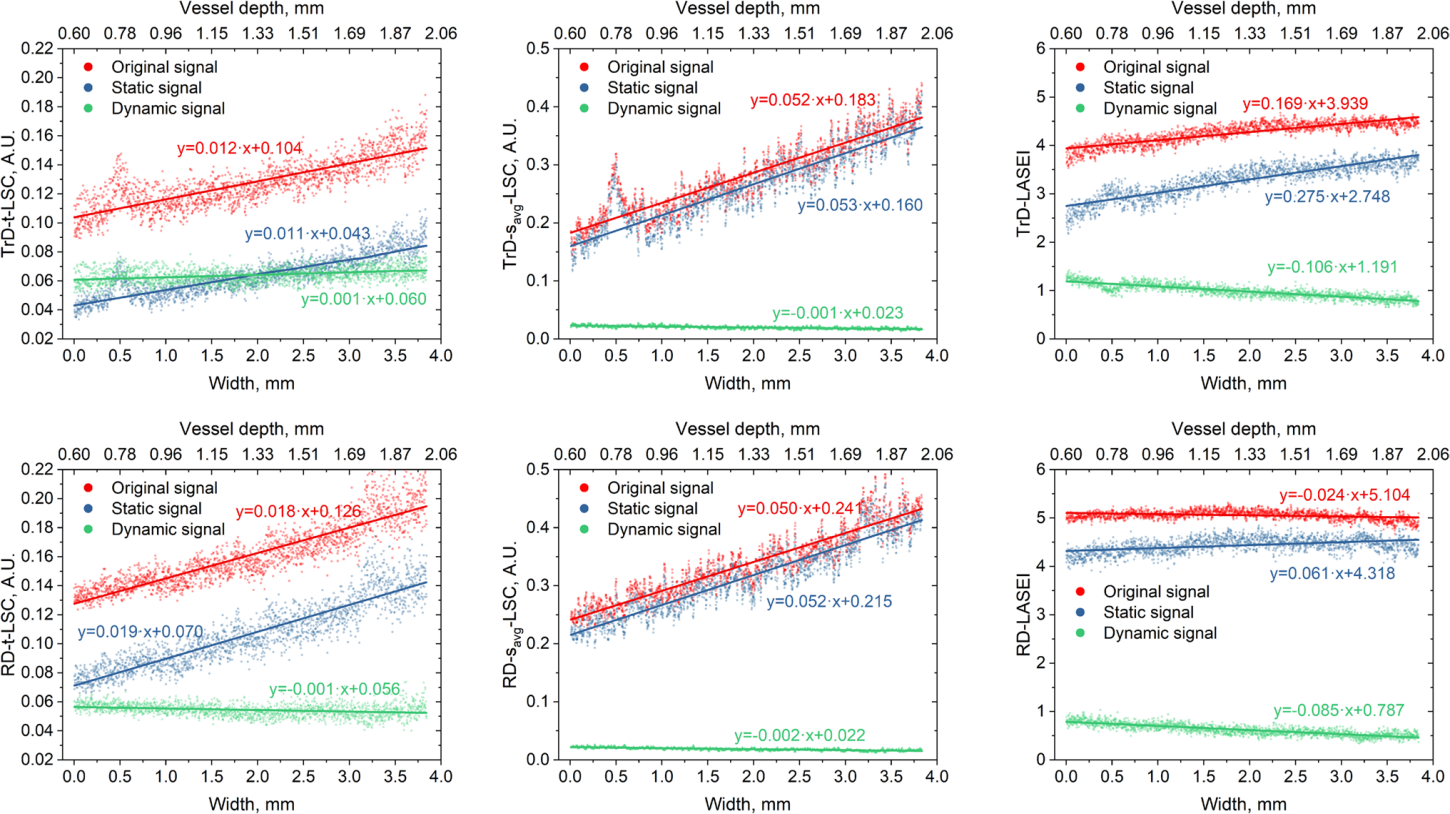
Averaged profiles of speckle contrast values in the central vessel region (ROI in Fig. 3) obtained using various laser speckle imaging (LSI) methods in transmissive (TrD) and reflective (RD) laser detection modes. The profiles are presented for RSI (Original signal), the *Static component* (Static signal), and the *Dynamic component* (Dynamic signal). The lower axis corresponds to the width of the visualization window, and the upper axis corresponds to the vessel depth. Linear approximation of the data are shown on the graphs with their corresponding equations

Additionally, as seen in Fig. 3, the speckle contrast and entropy values after processing RSI increase in the capillary region as the vessel depth increases. However, in the *Dynamic component* group, the speckle contrast values are almost independent of the thickness of the scattering layer above the vessel. This behavior is more clearly observed in Fig. 4.

Figure 4 shows the averaged profiles of speckle contrast values in the central vessel region (ROI in Fig. 3), obtained for various LSI methods in transmissive and reflective laser detection modes. The profiles are plotted as a function of the visualization window width (bottom axis) and vessel depth (top axis). The graphs display the results for RSI (*Original signal*), the *Static component* (Static signal), and the *Dynamic component* (Dynamic signal). The obtained data were approximated by a linear function, and the equations of these approximations are shown on the graphs. Results for the st-LSCI method are not shown in this example, as they are nearly identical to those for s_avg_-LSCI.

The main conclusion that can be drawn from the graphs in Fig. 4 is that after extracting the *Dynamic component*, the speckle contrast values become almost independent of the vessel depth, as confirmed by the horizontal approximation lines. Additionally, before applying PCA filtering, the speckle contrast values in the vessel region differed significantly between the TrD and RD modes for the same LSCI method. However, after extracting the *Dynamic component*, they become quite similar. At the same time, differences between the temporal and spatial speckle contrast values persist. It is also worth noting that PCA-based filtering did not improve the visualization results in LASEI, which may indicate that this method is less sensitive to the separation of static and dynamic signal components. The obtained results are in good agreement with the findings of [23], particularly in terms of minimizing the sensitivity to vessel depth.

### Comparison of LSI methods in TrD and RD modes

**Fig. 5:**
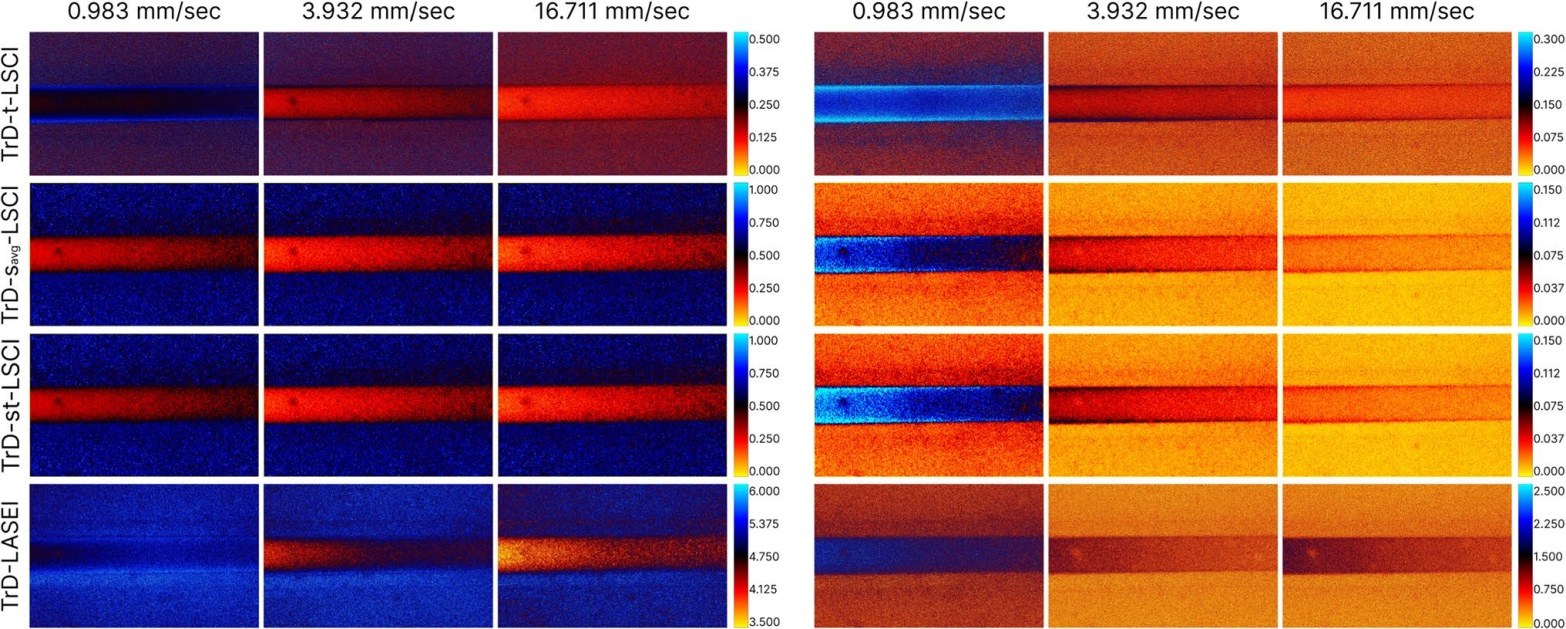
Color maps of speckle contrast obtained using various laser speckle imaging (LSI) methods in the transmissive detection mode (TrD) at different linear fluid velocities in the vessel (indicated above the images). The left columns show the results of traditional LSI methods, while the right columns present the results after extracting the dynamic signal component

Figure 5 demonstrates color maps obtained using various LSI methods in the TrD mode at different linear fluid velocities in the vessel, indicated above the images. The left columns represent the results of traditional LSI methods—*Original signal*, while the right columns show the results after extracting the *Dynamic component*. The rows from top to bottom correspond to different LSI methods: temporal laser speckle contrast imaging (t-LSCI), spatial laser speckle contrast imaging with time-averaging (s_avg_-LSCI), spatiotemporal laser speckle contrast imaging (st-LSCI), and speckle entropy imaging (LASEI). The color palette was chosen to effectively capture the dynamic range for each LSI method at different fluid velocities. Comparing the images before and after extracting the *Dynamic component* allows for an evaluation of the impact of fluid velocity on visualization results and shows an improvement in visualization quality after applying PCA filtering by minimizing the influence of the thickness of the scattering layer above the glass capillary—the speckle contrast values in the vessel region become more uniform compared to the results of traditional LSI.

**Fig. 6:**
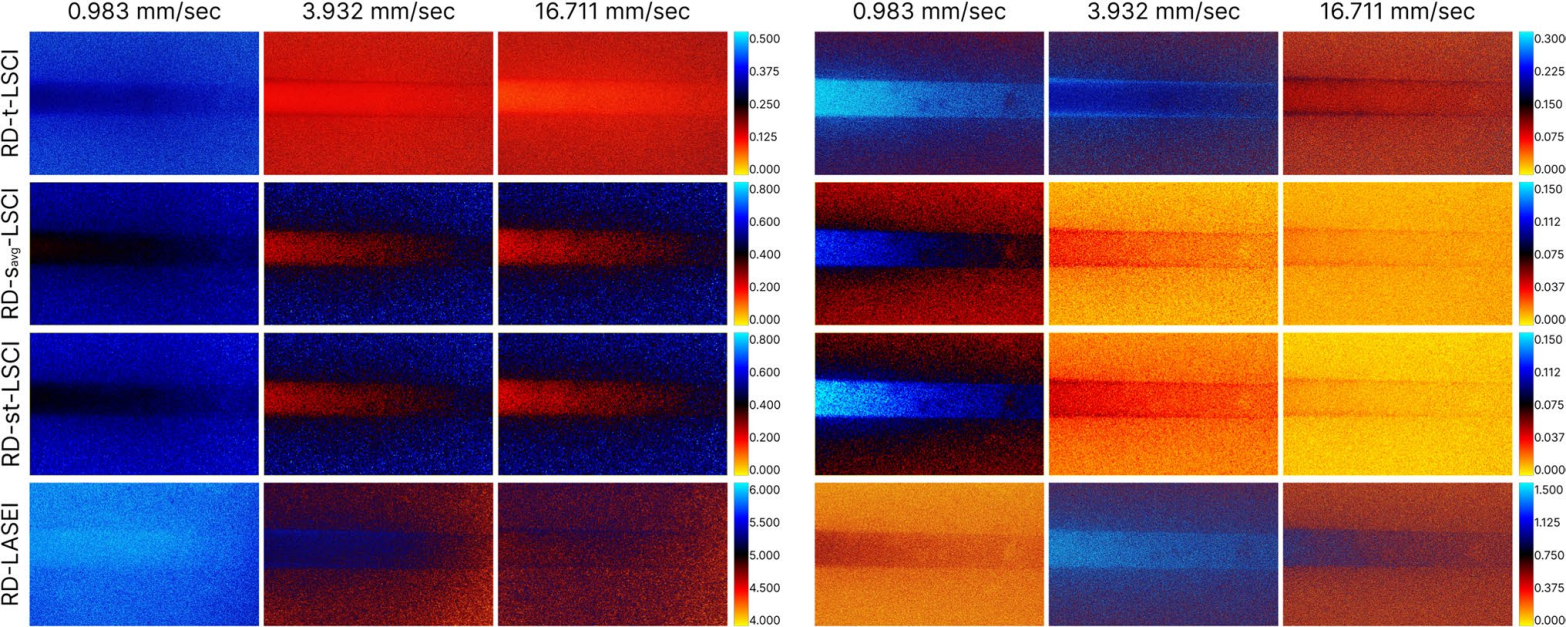
Color maps of speckle contrast obtained using various laser speckle imaging (LSI) methods in the reflective detection mode (RD) at different linear fluid velocities in the vessel (indicated above the images). The left columns show the results of traditional LSI methods, while the right columns present the results after extracting the dynamic signal component

Similar to Fig. 5, Fig. 6 presents color maps obtained using various laser speckle imaging (LSI) methods in the reflective detection mode (RD) at different linear fluid velocities in the vessel.

An observed decrease in speckle contrast values in the background region was noted for all LSCI methods as the flow velocity increased, particularly after PCA filtering. This observation requires further analysis and explanation, as it does not align with classical expectations for static regions. The reduction in speckle contrast values in the static background region with increasing flow velocity may be associated with phase changes occurring when radiation interacts with moving particles, such as erythrocytes or intralipid droplets. There is also a portion of the radiation that is scattered sideways after interacting with moving particles and reaches the detector matrix from the static background region as a result of multiple scattering. The phase changes depend on the velocity of the particles in the vessel. Thus, at low velocities, the phase changes are insignificant, and the influence on the background is minimal, whereas at high velocities, the phase changes become more pronounced, leading to a reduction in the background speckle contrast. It is important to note that changes in the scattering properties of both the static and dynamic regions can significantly alter this pattern. In addition, the scattering indicatrix (anisotropy) of erythrocytes and intralipid droplets may differ.

**Fig. 7:**
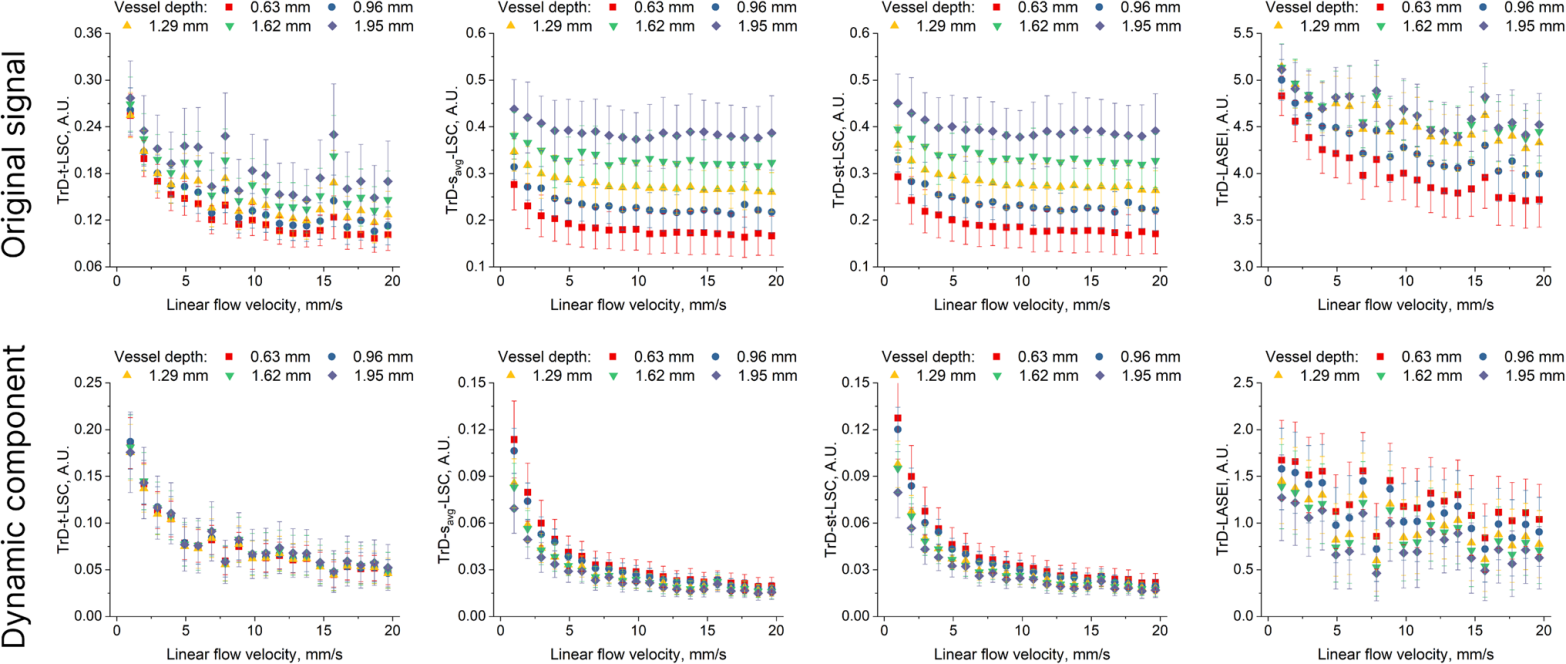
Dependence of speckle contrast and entropy values on the linear fluid velocity in the vessel for five ROI at different vessel depths, obtained in transmissive (TrD) detection mode. The graphs are presented for both the *Original signal* and the signal after extracting the *Dynamic component*

**Fig. 8:**
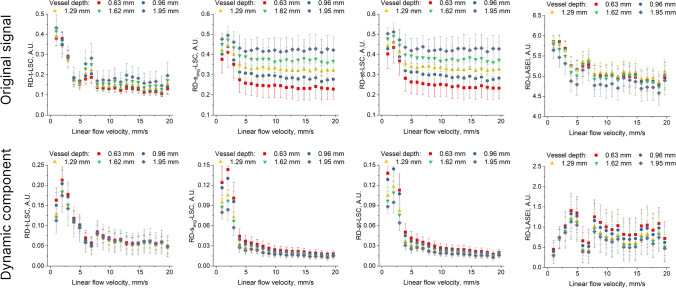
Dependence of speckle contrast and entropy values on the linear fluid velocity in the vessel for five ROI at different vessel depths, obtained in reflective (RD) detection mode. The graphs are presented for both the *Original signal* and the signal after extracting the *Dynamic component*

Figures 7 and 8 show a set of graphs depicting the dependence of speckle contrast and entropy values on the linear fluid velocity in the vessel, averaged for five equally spaced regions of interest (ROI) at different vessel depths. The regions of interest were rectangular areas measuring 100 µm × 100 µm, located in the central part of the vessel. The graphs are divided into two groups: for the TrD mode and the RD mode. Each group includes results for the *Original signal* and for the signal after extracting the *Dynamic component*. In both groups (TrD and RD), there is a significant difference in the dependence of speckle contrast and entropy values on fluid velocity between the *Original signal* and the signal after extracting the *Dynamic component*. However, the differences between TrD and RD modes are more pronounced before applying PCA filtering. After extracting the *Dynamic component*, the differences between TrD and RD become less noticeable, especially at fluid velocities greater than 5.6 mm/s, indicating that PCA filtering reduces the influence of the scattering layer on the measurements. For the *Original signal*, the speckle contrast and entropy values decrease with increasing fluid velocity, as expected, since higher velocity leads to reduced speckle contrast due to blurring caused by moving particles. Additionally, the graphs show that for the original signal, the speckle contrast and entropy values depend on the vessel depth, with deeper vessels exhibiting higher speckle contrast values. However, after extracting the *Dynamic component*, this dependence becomes less pronounced for both modes: TrD and RD.

Comparing LSCI methods with LASEI, it can be observed that the dependence of entropy values on the linear fluid speed exhibits more linear behavior throughout the entire range of investigated velocities. This aligns well with the main conclusions of studies [33, 36, 55]. As far as we know, LASEI in TrD mode has not been previously studied. In the context of this work, we observe greater sensitivity of the LASEI method in TrD mode to changes in the scattering fluid speed. However, the application of PCA filtering did not show improvements for LASEI, unlike the LSCI group of methods.

In the RD mode (Fig. 8), for the dynamic component, we observed a deviation from the theoretical prediction in the speckle contrast distribution at low flow rates. We speculate that this deviation arises from small changes in the speckle pattern at low flow rates, which become closer to the noise level. As a result, the PCA filtering algorithm may incorrectly attribute minor changes in the speckle pattern to the static components, leading to the observed discrepancies. This behavior is not observed in the TrD mode, suggesting that the signal formation mechanisms differ between the two modes. Monte-Carlo simulations performed in [19] support this speculation, showing that the scattered radiation intensity associated with the useful signal is higher in the TrD mode when the vessel depth exceeds 200 μm. In the context of our study, we speculate that at low flow rates, changes in the RSI are minimal. Given the relatively low intensity of laser radiation scattered from the vessel at a depth greater than 600 μm, we observe a deviation in the distribution of speckle contrast values from theoretical expectations when using RD-LSI with PCA filtering.

### Effect of vessel depth on LSI results

**Fig. 9:**
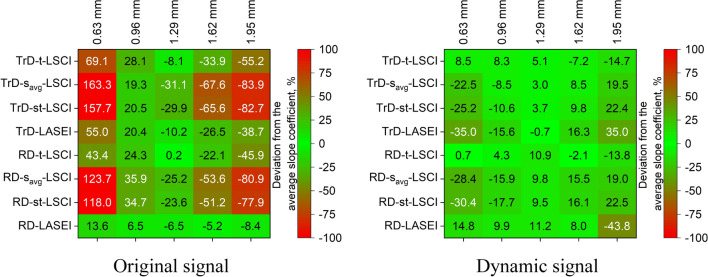
Matrices of the percentage deviation of the slope coefficient of *SFI*(*v*) from the average value for each laser speckle imaging (LSI) method depending on vessel depth. The left image shows the results for the *Original signal*, while the right image shows the results after extracting the *Dynamic component*. A significant deviation of the slope coefficient indicates a high dependence of the method on vessel depth and, consequently, its lower reliability

Figure 9 shows matrices illustrating the percentage deviation of the slope coefficient of *SFI*(*v*) from the average value (averaged across all depths) for each LSI method depending on vessel depth. The left image shows the results for the *Original signal*, while the right image shows the results after extracting the *Dynamic component* via PCA filtering. For the *Original signal*, it is noticeable that all LSI methods show significant sensitivity to the depth of the glass capillary. However, the TrD-LASEI and RD-LASEI methods demonstrate more stable results, with smaller deviations from the average slope of *SFI*(*v*), indicating their greater stability across all depths. After applying PCA filtering, it is evident that deviations significantly decreased for all LSCI methods. For example, the t-LSCI method in both TrD and RD modes shows the least sensitivity to vessel depth, indicating improved reliability of these methods after extracting the *Dynamic component*.

### Impact of flow velocity on LSI results

**Fig. 10:**
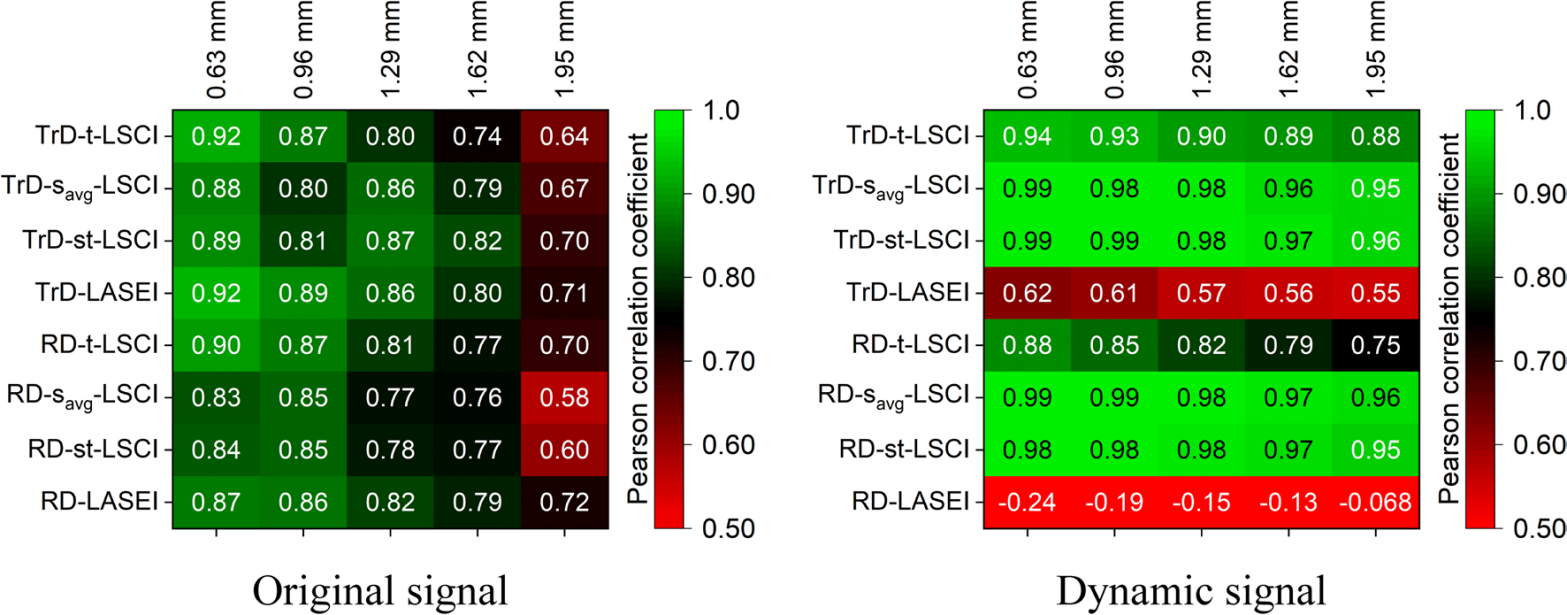
Matrices of Pearson linear correlation coefficients between the *SFI*(*v*) parameter and the actual fluid velocity for various laser speckle imaging (LSI) methods and different vessel depths. The left image shows the correlation coefficients for the original signal, while the right image shows the coefficients after extracting the *Dynamic component*. High correlation values indicate high sensitivity of the method to changes in fluid velocity, while low correlation values suggest reduced sensitivity

Figure 10 presents matrices of Pearson linear correlation coefficients between the *SFI* parameter and the actual fluid speed for various LSI methods and different vessel depths. The left image shows the correlation coefficients for the *Original signal*, while the right image shows the coefficients after extracting the *Dynamic component*. For the *Original signal*, LSI methods generally show high Pearson correlation coefficients, especially for shallow vessels (0.63 and 0.96 mm), indicating good sensitivity to fluid speed at shallow depths. However, as vessel depth increases, the correlation coefficients decrease, indicating reduced sensitivity of the methods. For example, the TrD-t-LSCI method shows a high correlation coefficient (0.92) at a depth of 0.63 mm, but this value drops to 0.64 at a depth of 1.95 mm. The smallest decrease in LSI method sensitivity is observed with RD-LASEI, where the correlation decreases from 0.87 to 0.72. After applying PCA filtering, improvement in correlation is observed for some methods, especially for s_avg_-LSCI and st-LSCI methods in both TrD and RD modes, which show high correlation across all depths, indicating increased sensitivity to fluid velocity. For example, the TrD-st-LSCI method shows a correlation close to 1.0 at all depths, indicating high reliability of the method after extracting the *Dynamic component*. On the other hand, TrD-LASEI and RD-LASEI methods show a decrease in correlation after filtering, especially at greater depths, which may indicate their reduced effectiveness after PCA filtering.

### In vivo demonstration of LSI combined with PCA

**Fig. 11:**
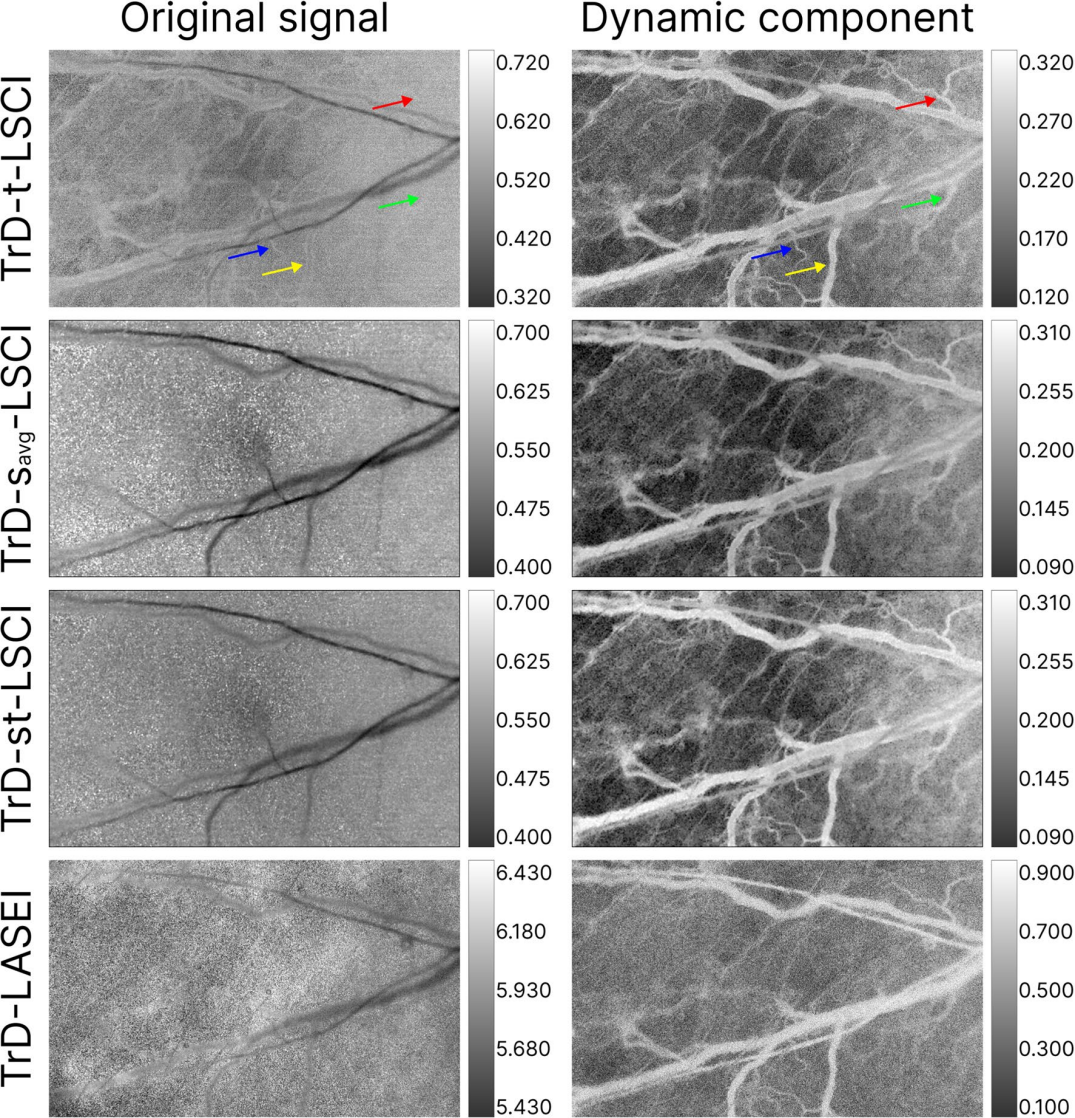
Laser speckle contrast and speckle entropy images of a laboratory mouse's ear before (*Original signal*) and after PCA filtering (*Dynamic component*) in the transmissive detection mode (TrD). The LSI methods are indicated on the left. Colored arrows indicate certain vessels whose images differ significantly before and after PCA

Figure 11 shows the results of in vivo imaging of a laboratory mouse's ear using various LSI methods in the transmissive detection mode (TrD). The left column shows images obtained for the *Original signal*, while the right column shows images obtained after extracting the *Dynamic component*. The LSI system parameters corresponded to those used in phantom studies. In the traditional LSI images (left column), vessels are visible, but artifacts and noise are also present, especially in areas without vessels. After extracting the *Dynamic component* of the speckle signal, the images become clearer, and the vessels stand out against a more homogeneous background. The vessels become more distinguishable, while background noise and artifacts are significantly reduced. Colored arrows indicate certain vessels that are barely visible with traditional LSI processing. PCA filtering improved vascular imaging for all studied methods.

**Fig. 12:**
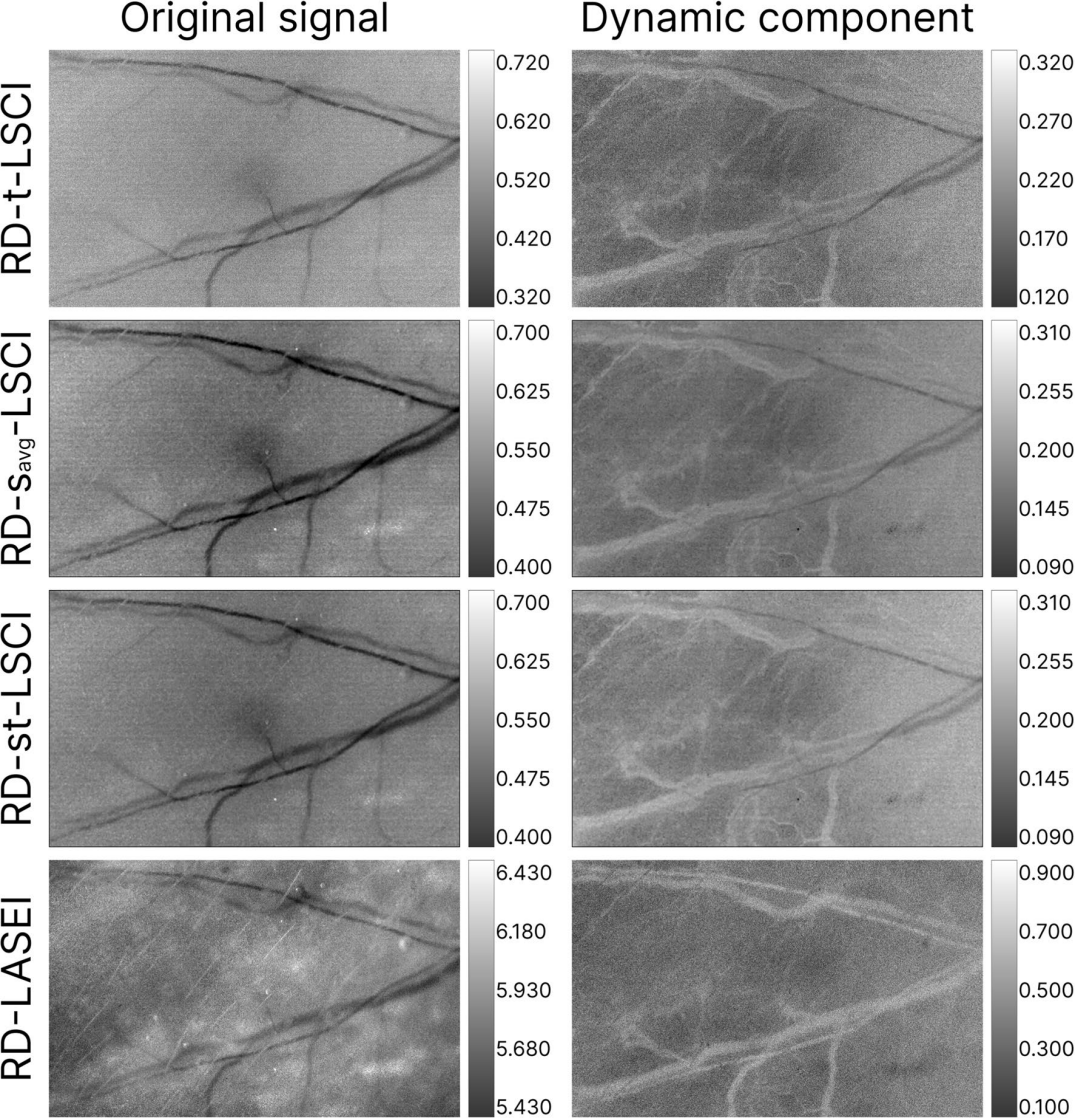
Laser speckle contrast and speckle entropy images of a laboratory mouse's ear before (*Original signal*) and after PCA filtering (*Dynamic component*) in the reflective detection mode (RD). The LSI methods are indicated on the left

Similar to Fig. 11, Fig. 12 presents the results of LSI processing with and without PCA filtering for the RD mode. The capture area was the same as in the TrD mode. In the comparison of RD and TrD modes, PCA filtering also improved vascular imaging for all studied methods. However, images in the TrD mode appear sharper and more contrasted.

Similar to phantom studies, in LSI images in Figs. 11 and 12 for the dynamic component, low speckle contrast and entropy values are observed in areas between vessels, associated with static tissue. This effect is due to the fact that in the dynamic component group, the signal from static scatterers in the surrounding tissue is either absent or minimized.

The results of mouse ear image processing demonstrate the potential of applying PCA filtering in combination with various LSI methods to improve the visualization of vascular structures in vivo. Phantom studies with varying vessel depths showed an increase in the sensitivity of LSCI methods to changes in fluid flow velocity and a minimization of sensitivity to vessel depth. The obtained results confirm and complement the findings of [23], particularly in terms of enhancing vascular visualization by separating static and dynamic components of the speckle signal through PCA filtering. These methods may be useful for diagnostics and research in biomedicine, where high image accuracy, reliability and contrast are required.

The current results confirm the reproducibility and reliability of the method, demonstrating its robustness in repeated measurements. This validation represents an important step before expanding the study to include a larger number of samples, where biological variability will be further investigated.

## Conclusion

This study proposed and investigated a method for improving the quality of vascular visualization using laser speckle contrast and speckle entropy imaging combined with PCA filtering. The results demonstrated that PCA application significantly enhances the contrast and clarity of vascular images by minimizing the influence of static scatterers, such as surface tissues. Studies on optical phantoms confirmed that the method effectively separates static and dynamic components of the speckle signal, while also increasing the sensitivity of the techniques to blood flow velocity and reducing their dependence on vessel depth. Which is in good agreement with the results of other studies [23, 37, 38]. For LSCI methods with PCA filtering, the Pearson correlation coefficient between the *SFI*(*v*) parameter and the linear fluid velocity increased across the entire range of vessel depths (from 0.63 to 1.95 mm). On average, for TrD-LSCI with PCA filtering, the correlation coefficient increased from 0.80 to 0.95, and for RD-LSCI—from 0.78 to 0.92, corresponding to increases of 18.7% and 17.9%, respectively. The highest correlation coefficient was observed for TrD-st-LSCI combined with PCA—0.99 at a depth of 0.63 mm, decreasing to 0.96 at a depth of 1.95 mm. Meanwhile, LASEI combined with PCA did not show improvements. However, LASEI without PCA filtering demonstrated a higher correlation with linear velocity compared to LSCI methods without PCA—0.83 versus 0.80 for TrD and 0.81 versus 0.78 for RD modes, respectively.

In vivo studies on a laboratory mouse ear confirmed the effectiveness of the proposed approach, providing significantly more detailed and contrasted images of the vascular network.

These results highlight the method's ability to assess blood flow velocity independently of vessel depth, overcoming one of the key limitations of traditional LSI methods.

The proposed approach holds great potential for non-invasive biomedical imaging, offering improved diagnostic precision, reliability, and contrast in vascular visualization. These findings could be particularly valuable for advancing the use of LSI in clinical diagnostics and biomedical research, where high accuracy in blood flow monitoring is essential.

In the future, the PCA filtering method combined with LSI may expand the capabilities of LSCI for visualizing blood flow through scattering tissue layers, which is particularly important in situations where direct access to vessels is limited.

## Data Availability

The data that support the findings of this study are available from the corresponding author, upon reasonable request.
